# Engineering Calreticulin-Targeting Monobodies to Detect Immunogenic Cell Death in Cancer Chemotherapy

**DOI:** 10.3390/cancers13112801

**Published:** 2021-06-04

**Authors:** Ying Zhang, Ramar Thangam, Sung-Hwan You, Rukhsora D. Sultonova, Akhil Venu, Jung-Joon Min, Yeongjin Hong

**Affiliations:** 1 Department of Nuclear Medicine, Institute for Molecular Imaging and Theranostics, Hwasun Hospital, Chonnam National University Medical School, Hwasun 58128, Korea; zhangyingneu@outlook.com (Y.Z.); thangam1985@yahoo.co.in (R.T.); yshyoou@naver.com (S.-H.Y.); ruxsora.sultonova@gmail.com (R.D.S.); akhilvenu.1@gmail.com (A.V.); 2 Department of Materials Science & Engineering, Korea University, Seoul 02841, Korea; 3 Department of Microbiology, Chonnam National University Medical School, Hwasun 58128, Korea

**Keywords:** monobody, calreticulin, immunogenic cell death (ICD), optical bioluminescence imaging, early prediction of chemotherapeutic response

## Abstract

**Simple Summary:**

Cancer cell surface–exposed calreticulin (ecto-CRT) is the primitive form of signal during immunogenic cell death (ICD). It is a well-known candidate to allow “eat-me” signal from dying cells, which further contributes to their perception in directing the immune system. Various forms of anticancer agents and ionizing radiation can facilitate the ICD via ecto-CRT exposure. We engineered CRT-specific human fibronectin domain III (FN3) monobodies (FN3-CRT-Rluc8) fused with peptide sequences for CRT imaging. We assessed the theragnostic use of engineered monobodies for ecto-CRT imaging during ICD for early therapeutic prediction response. Our findings demonstrated that engineered monobodies could involve in targeting dying cells via anticancer-related immunogenic chemotherapeutic treatments, and the obtained imaging results could be used to detect pre-apoptotic cells in ICD. Our data provides the novel FN3-based ecto-CRT imaging method and enables the early immuno-therapeutic response predictions, thereby facilitating early determinations in cancer chemotherapies.

**Abstract:**

Surface-exposed calreticulin (ecto-CRT) plays a crucial role in the phagocytic removal of apoptotic cells during immunotherapy. Ecto-CRT is an immunogenic signal induced in response to treatment with chemotherapeutic agents such as doxorubicin (DOX) and mitoxantrone (MTX), and two peptides (KLGFFKR (Integrin-α) and GQPMYGQPMY (CRT binding peptide 1, Hep-I)) are known to specifically bind CRT. To engineer CRT-specific monobodies as agents to detect immunogenic cell death (ICD), we fused these peptide sequences at the binding loops (BC and FG) of human fibronectin domain III (FN3). CRT-specific monobodies were purified from *E. coli* by affinity chromatography. Using these monobodies, ecto-CRT was evaluated in vitro, in cultured cancer cell lines (CT-26, MC-38, HeLa, and MDA-MB-231), or in mice after anticancer drug treatment. Monobodies with both peptide sequences (CRT3 and CRT4) showed higher binding to ecto-CRT than those with a single peptide sequence. The binding affinity of the Rluc8 fusion protein–engineered monobodies (CRT3-Rluc8 and CRT4-Rluc8) to CRT was about 8 nM, and the half-life in serum and tumor tissue was about 12 h. By flow cytometry and confocal immunofluorescence of cancer cell lines, and by in vivo optical bioluminescence imaging of tumor-bearing mice, CRT3-Rluc8 and CRT4-Rluc8 bound specifically to ecto-CRT and effectively detected pre-apoptotic cells after treatment with ICD-inducing agents (DOX and MTX) but not a non-ICD-inducing agent (gemcitabine). Using CRT-specific monobodies, it is possible to detect ecto-CRT induction in cancer cells in response to drug exposure. This technique may be used to predict the therapeutic efficiency of chemo- and immuno-therapeutics early during anticancer treatment.

## 1. Introduction

Immunogenic cell death (ICD) is a form of apoptotic cell death resulting in a regulated activation of the immune response and is generally caused by endoplasmic reticulum (ER) stress–associated apoptosis [1]. Calreticulin (CRT) is a chaperone with a molecular weight (MW) of 46 kDa that is abundantly localized to the ER lumen, highly bound with Ca^2+^, and involved in various cellular processes associated with Ca^2+^ signaling in the control of cellular responses [2,3]. Mammalian CRT proteins in various species are highly conserved [4,5]. CRT exposed on the cell surface of the plasma membrane (ecto-CRT) is a primitive pro-phagocytic signaling protein [6]. The cell surface expression of ecto-CRT is highly induced by chemo-, radio-, and ablative immunotherapies [7,8]. Expression of ecto-CRT serves as an “eat-me” signal on the cell surface, resulting in ICD. Immunogenic antitumor agents including doxorubicin (DOX) and mitoxantrone (MTX) induce ICD via ecto-CRT [9,10,11,12,13,14,15,16]. Conversely, gemcitabine (GEM), a non-immunogenic drug, does not result in the translocation of CRT to the plasma membrane and fails to induce ICD [17,18,19,20]. Most importantly, ecto-CRT can also be used as a marker for the prediction of early therapeutic responses to anticancer agents [8,10,19], which has a significant impact on the development of cancer immunotherapies.

Fibronectin (FN) is a high MW (~500 kDa) glycoprotein found in plasma and extracellular matrix (ECM) and specifically binds to integrins associated with membrane-spanning receptor proteins [21,22]. FN contains three types of repeated domains, including an arginine–glycine–aspartic acid (RGD) motif that is related to signaling events occurring via integrin activation [23,24]. The structure of the FN type III domain (FN3) is well defined [25]. This domain is a monomeric structure with seven antiparallel β-sandwiches and three loops (BC, DE, and FG) with variable length and sequence [26,27,28]. This domain is found in various proteins in animals and is structurally similar to immunoglobulin-like regions of antibodies. This domain has become one of the most widely used biological scaffolds to generate new binding proteins (monobodies) [25,29]. As scaffolds for artificial binding proteins, monobodies can be used to overcome many of the disadvantages of antibodies, such as their large molecular size and structural complexity [30,31]. Among them, the VEGFR2-targeted monobody CT-322 has been tested in phase 2 clinical trials [32], but showed poor efficacy in recurrent glioblastoma possibly due to a lack of clinical effect from inhibiting VEGFR2 [33]. In spite of these clinical failures, researchers have shown continued interest in developing safe monobodies [32,34,35]. These results validate the use of the FN3 domain as a protein-binding scaffold.

Luciferase proteins of photo-proteins family can be found in species of marine organisms, prokaryotes, and some insects. Green bioluminescent Rluc8 protein/enzyme is normally found in *Renilla luciferase* and *Renilla reniformis*, which has a wider bioimaging application as reporters in prokaryotes and eukaryotes [36]. The bioluminescence of this Rluc8 is generated via catalyzation of coelenterazine oxidation, thereby releasing the blue light in a broad range at 480 nm through imidazolopyrazine structure [37,38]. Reports stated that Rluc8 can be used as a reporter protein to monitor the gene expression in both in vitro and in vivo models [39,40,41,42]. Thus, we chose this Rluc8 as a reporter protein of the study and demonstrated in vivo bioluminescence imaging potentials of our CRT monobodies.

Lipoprotein receptor–related protein (LRP) is the signaling co-receptor for ecto-CRT. It consists of two noncovalently attached subunits. LPR binds to the NH_2_-terminal heparin-binding domain of CRT [43]. Previously, a CRT-targeting synthetic peptide, Int-α (KLGFFKR), was reported, and its dissociation constant (Kd) against CRT was 1.868 µM [8,44,45]. The CRT-binding peptide, Hep-I (GQPMYGQPMY), was also reported to target to CRT with an efficiency of 34% and 11% in the solid and soluble phases, respectively [43,46,47]. Although CRT-targeting peptides were easily identified from various library screens and chemically synthesized, their low affinities have restricted the in vivo application.

**Scheme 1:**
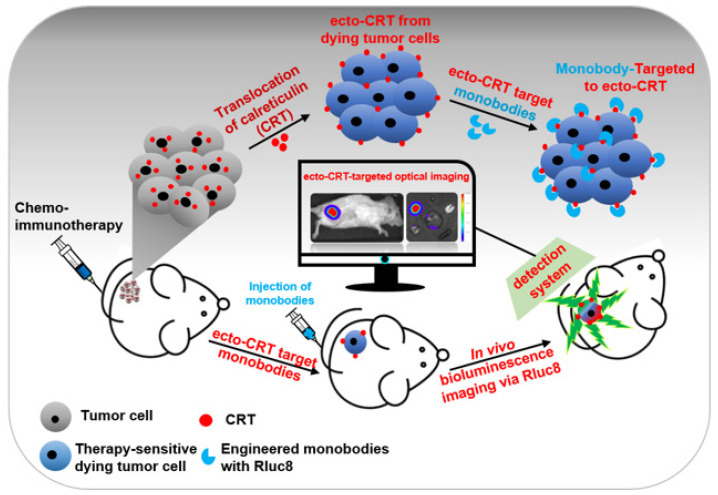
Schematic representation of the early detection of immunogenic cell death (ICD) in cultured tumor cells and mouse models. After anticancer drug treatment, calreticulin (CRT) translocate from the endoplasmic reticulum to the plasma membrane of tumor cells (ecto-CRT). Ecto-CRT is detected with the injected Rluc8-fused CRT monobodies, and the target binding is assessed using a bioluminescence imaging system. The luminescent signal represents ICD after chemotherapy.

In this report, we aimed to generate highly specific CRT-targeting monobodies using the structurally immobilized CRT-targeting peptides Int-α and Hep-I. After engineering the monobodies by grafting these peptide sequences at their binding loops (BC and FG), we characterized them for ecto-CRT targeting efficiency in vitro and in vivo after treatment of tumor-bearing mice with immunogenic and non-immunogenic anticancer agents (Scheme 1).

## 2. Materials and Methods

### 2.1. Chemical, Reagents, and Cell Lines

The recombinant rabbit calreticulin protein (rCRT, ab15729) was purchased from Abcam (Cambridge, UK). Mouse colon (CT-26 and MC-38), human cervix (HeLa), and metastatic breast (MDA-MB-231) cancer cell lines were purchased from the American Type Culture Collection (ATCC, Manassas, VA, USA). The cells were cultured in DMEM media (Sigma-Aldrich, Saint Louis, MA, USA) supplemented with 10% fetal bovine serum (Gibco/Thermo Fisher Scientific, Waltham, MA, USA), 100 IU penicillin/streptomycin solution (Sigma-Aldrich, Saint Louis, MA, USA), and non-essential amino acids (Sigma-Aldrich). Phosphate-buffered saline (PBS) was purchased from Invitrogen/Thermo Fisher Scientific (Waltham, MA, USA). ELISA kits (coating buffer, ELISA/ELISPOT diluent, pre-titrated avidin-horseradish peroxidase (HRP), and TMB solution) were purchased from Invitrogen/Thermo Fisher Scientific. Various antibodies used in this study were purchased from Abcam and Thermo Fisher Scientific. All other chemicals for cloning and molecular biology experiments were purchased from Thermo Fisher Scientific unless otherwise specified. All transformations and plasmid purifications during cloning were performed using *E. coli* DH5α (Enzynomics, Daejeon, Korea) cultured in LB medium and selected with kanamycin (50 µg/mL). pETh-FN3(DGR) (named #DGR), an expression vector for FN3 with the RGD sequence mutated to DGR, was described, and it was expressed and purified as a negative control for the monobodies [48,49].

### 2.2. Construction of Monobody Expression Vectors

Among the three loops within FN3 for target binding, two loops (BC and FG) have a much longer length for sequence grafting [50,51]. We replaced these wild-type loop sequences with Hep1 and Int-α peptides to construct the monobody genes. Using these sequences, we designed polymerase chain reaction (PCR) primers. Each loop fragment was amplified using the KOD plus PCR kit (Toyobo, Osaka, Japan). Primer sets for BC loops with Hep-1 and Int-α were termed BC-F: BC-Hep-1 and BC-F: BC-Int-α, respectively. The DE loop was amplified with DE-F:DE-R primer sets. Primer sets for FG loops with Hep-1 and Int-α were FG-Hep-1: 94 old-R and FG-Int-α: 94 old-R, respectively. After agarose gel separation and purification of PCR fragments, monobody genes were amplified with the 94 old-F: 94 old-R primer set against a mixture of BC, DE, and FG fragments. The amplified fragments were digested with *Nhe*1 and *Bam*H1 enzymes and cloned into the same sites of the pETh vector [52]. The resulting plasmids were named according to insertion peptide sequences in FG and BC loops as pETh-CRT1, pETh-CRT2, pETh-CRT3, pETh-CRT4, pETh-CRT5, and pETh-CRT6, respectively (abbreviated as CRT1–CRT6).

**Figure 1:**
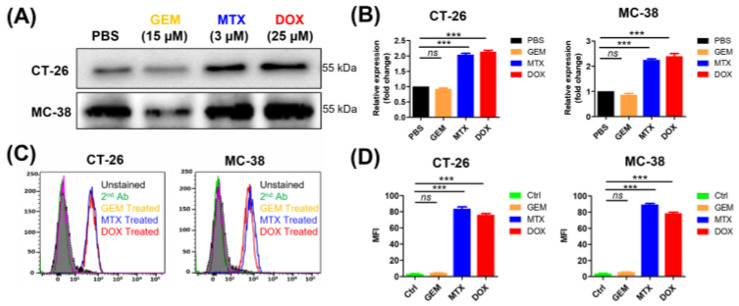
Measurement of ecto-CRT during immunogenic cell death (ICD) in cancer cells after anticancer drug treatment. Cancer cells lines, including CT-26 and MC-38, were treated with anticancer drugs at the indicated concentrations for 4 h and ecto-CRT (CRT translocated from the ER to the plasma membrane) was measured during the pre-apoptotic stage. (**A**) Western blot analysis. The membrane fractions were isolated and separated by SDS-PAGE. Ecto-CRT was detected with an anti-CRT antibody. (**B**) Quantitation of Western blot analysis. The ecto-CRT detected in (**A**) was quantitated and the relative levels are shown. (**C**) Flow cytometry analysis of ecto-CRT. Cells were treated with anticancer drugs and stained with an anti-CRT antibody. (**D**) The mean fluorescence intensities (MFIs) of the samples shown in (**C**) were measured and the relative levels were plotted. Second Ab is used as control of the background fluorescence signals. Data represent the mean ± standard error (*n* = 3). *** *p* < 0.001 and *ns* = non-significant. (MTX, mitoxantrone; DOX, doxorubicin; GEM, gemcitabine).

CRT3 and CRT4 monobody genes fused with the Rluc8 reporter gene were constructed with the exponential mega-priming (EMP) PCR method [53]. First, the monobody megaprimers were amplified against the pETh-CRT plasmid with the T7-F: FG-R primer set for 30 cycles. After purification in agarose gels, EMP PCR was performed with the T7-F: T7-R primer set using a mixture of the megaprimers and pETh-E1-Rluc8 [52]. The amplified PCR fragments were phosphorylated with polynucleotide kinase, digested with *Dpn*I, and ligated with T4 DNA ligase. Finally, they were transformed into *E. coli* DH5α competent cells. The resulting plasmids were pETh-CRT3-Rluc8 (CRT3-Rluc8) and pETh-CRT4-Rluc8 (CRT4-Rluc8), respectively. All primers used in this study are listed in Table S1. All plasmids constructed in this study were confirmed through sequencing analysis (Figure 1A).

### 2.3. Purification of Monobodies

Production of recombinant monobody (CRT-Rluc8) in *E. coli* BL21(DE3) strain (Invitrogen/Thermo Fisher Scientific). After transformation with the plasmids described above, bacteria were cultured in LB broth containing kanamycin (50 µg/mL). Bacteria were cultured overnight, and 10 mL of the culture was used to inoculate 1 L of fresh LB broth and grown at 37 °C. At an optical density at 600 nm (OD_600_) of 0.6, 1 M isopropyl-β-D-thiogalactopyranoside (IPTG) was added to a final concentration of 0.5 mM, and the bacteria were further cultured at 37 °C for 4 h. Bacteria pellets were harvested by centrifugation (8000× *g* at 4 °C). The pellets were re-suspended in ice-cold lysis buffer (50 mM NaH_2_PO_4_, 300 mM NaCl, and 10 mM imidazole; pH 8.0) containing 100 μg/mL of lysozyme solution and further incubated for 30 min on ice. After gentle sonication on ice, bacteria were centrifuged at 12,000× *g* for 20 min at 4 °C, and the supernatants were collected. Large-scale monobody purification was done with His GraviTrap affinity columns (GE Healthcare, Chicago, IL, USA) using these supernatants, and the excess imidazole after affinity chromatography was removed using a PD-10 desalting column (GE Healthcare, Chicago, IL, USA).

### 2.4. Western Blot Analysis

Monobodies expressed in bacteria were detected by Western blot analysis. Original western blots can be found at Figure S11.The bacterial pellets or purified proteins were separated with 10% sodium dodecyl sulfate–polyacrylamide gel electrophoresis (SDS-PAGE) and transferred to nitrocellulose (NC) or polyvinylidene fluoride (PVDF) membranes (Bio-Rad, Des Plaines, IL, USA). The membranes were soaked in 5% skim milk in Tris-buffered saline containing 0.1% Tween 20 (TBS-T) for 1 h and then incubated with a mouse anti-His tag monoclonal antibody (1:1000 dilution; Abcam) for 4 h at room temperature. After washing three times with TBS-T, the membranes were incubated with an HRP-conjugated goat anti-mouse IgG antibody (1:5000 dilution; Abcam) for 1 h at room temperature. The His-tagged proteins were visualized using an enhanced chemiluminescence (ECL) Western blotting luminol reagent (Thermo Fisher Scientific) and recorded using the LAS-3000 chemiluminescence detection system (Fuji, Osaka, Japan).

To detect ecto-CRT on the plasma membrane, cancer cells were treated with anticancer agents [GEM (15 μM), MTX (3 μM), and DOX (25 μM)] for 4 h at 37 °C. Pre-apoptotic cells were collected and the proteins on the cell surface were isolated using the Plasma Membrane Protein Extraction Kit (Abcam). After the extracted proteins in the plasma membrane were quantified by bicinchoninic acid (BCA) assay, they were separated using 10% SDS-PAGE and transferred to PVDF membranes. The membranes were incubated with a CRT-specific primary antibody (1:1000 dilution; Abcam) overnight at 4 °C, followed by incubation with an HRP-conjugated secondary antibody (1:5000 dilution; Invitrogen/Thermo Fisher Scientific) for 30 min at room temperature. The ecto-CRT levels in drug-treated cells were measured and the relative levels were calculated by normalization to the levels in PBS-treated control cells using intensity ratio of each band via applying the densitometric quantifications [8,14].

### 2.5. Measurement of Binding Affinity

The binding affinities of Rluc8-fused CRT monobodies were measured by enzyme-linked immunosorbent assay (ELISA), as described previously [54,55]. At first, monobodies (0–10 μM) were added to 96-well microtiter plates (Corning, NY, USA). After incubation overnight at 4 °C, the wells were aspirated to remove the monobodies and washed four times with 250 µL PBS containing 1% Tween 20 (PBS-T) using an automated plate washer. Then, 200 µL of 1× ELISA/ELISPOT diluent buffer (Invitrogen/Thermo Fisher Scientific) was added to the wells. After incubation at room temperature for 1 h, the buffer was removed by aspiration and the wells were washed three times with PBS-T. Next, 100 µL of 10 µM rCRT in 1× ELISA/ELISPOT diluent buffer was added, and the plates were incubated at room temperature for 2 h and washed three times. To detect rCRT bound to monobody, 100 µL of rabbit anti-CRT antibody (1:500 dilution in 1× ELISA/ELISPOT buffer) was added to the wells. After incubation at room temperature for 2 h, the wells were washed three times with PBS-T, and 100 µL of biotin-conjugated anti-rabbit antibody (1:1000 dilution; Invitrogen/Thermo Fisher Scientific) was added. After incubation at room temperature for 1 h, the wells were washed five times with PBS-T. Finally, 100 µL of avidin-HRP (1:8000 dilution; Invitrogen/Thermo Fisher Scientific) was added to each well and the plates were incubated at room temperature for 30 min. Plates were then dried using absorbent paper to remove any residual buffer. After washing, 100 µL/well of TMB solution (Invitrogen/Thermo Fisher Scientific) was added to each well and the plates were incubated at room temperature for 15 min. To stop the color reaction, 50 µL/well of stop solution (0.5 M H_2_SO_4_) was added. The color intensity at OD_450_ was measured on a microtiter plate reader.

### 2.6. Confocal Immunofluorescence Imaging Analysis of Ecto-CRT

The cultured cancer cells were grown on a glass coverslip for 48 h and treated with anticancer agents (15 µM GEM, 3 µM MTX, or 25 µM DOX) for 4 h [8]. The cells were fixed with cold acetone for 10 min and incubated with 1% bovine serum albumin (BSA) in PBS for 10 min. Then, the cells were incubated with a mouse anti-CRT antibody (1:1000 dilution; Abcam) for 2 h on ice. After washing with PBS five times, the cells were incubated with an Alexa Fluor 488-conjugated anti-mouse IgG (H + L) Cross-Adsorbed antibody (1:2000 dilution; Thermo Fisher Scientific) for 1 h on ice and washed five times. To stain cell membranes, the cells were also incubated with Alexa Fluor 555-conjugated wheat germ agglutinin (WGA) (1:5000 dilution; Thermo Fisher Scientific). Finally, the cells were mounted using DAPI anti-fade mounting solution (Thermo Fisher Scientific). The fluorescence signals were imaged using an LSM510 confocal microscope and analyzed with ZEN-LSM imaging software (ZEISS, Jena, Germany).

### 2.7. Flow Cytometry Analysis

To measure the competitive binding between rCRT protein and cellular ecto-CRT, flow cytometry was performed as described [56,57,58]. Briefly, the cells were treated with DOX for 4 h. The cells detached from dishes (1 × 10^5^) were mixed with 10 µM rCRT. To these mixtures, monobodies were added (100 nM) and the cells were incubated for 1 h on ice. After washing with PBS, the cells were sequentially stained with a monoclonal anti-His tag monoclonal antibody (1:500 Abcam) and an Alexa Fluor 488-conjugated secondary antibody (Thermo Fisher Scientific) on ice for 1 h.

### 2.8. Monobody Stability Assay

To measure monobody stability in serum, 150 μL of Rluc8-fused monobody in PBS (60 μg) was mixed with an equal volume of mouse blood serum and incubated at 37 °C for various incubation times on 96-well black plates (Thermo Fisher Scientific). The bioluminescence activity was measured after adding 10 µL of 40 µg/mL coelenterazine (Biotium, Fremont, CA, USA) using an Orion L Microplate Luminometer (Berthold Detection Systems, Pforzheim, Germany) and an Infiniti M200 laser scanner (Tecan, Männedorf, Switzerland). Heat-inactivated serum was used as a negative control.

### 2.9. Mouse Models of Anticancer Therapy

BALB/c and C57BL/6 mice (female, 6 weeks old) were purchased from the Orient Company (South Korea). The animal studies reported here were done in accordance with the general principles and procedures outlined in the National Institutes of Health (NIH) Guidelines for the Care and Use of Animals [59], and all protocols were approved by the Animal Care and Use Committee of Chonnam National University (permit number: HCRL 16–001). BALB/c and C57BL/6 mice (*n* = 9 per group) were subcutaneously implanted with CT-26 or MC-38 tumor cells (1 × 10^6^ cells in 100 µL PBS) on the right flank. When the tumor size reached approximately 100 mm^3^, the anticancer agents (GEM, 15 mg/kg; DOX, 10 mg/kg; MTX, 2 mg/kg) were intraperitoneally administered to the mice three times in 2-day intervals. The therapeutic effects of the anticancer agents were measured based on changes in the tumor volumes and weights [8].

### 2.10. In Vivo and Ex Vivo Optical Bioluminescence Imaging Using CRT-Binding Monobodies

In vivo optical bioluminescence imaging analysis using Rluc8-fused CRT monobodies was performed before and after anticancer drug treatment. In brief, 2 days after the last administration of anticancer agents, monobodies (60 μg in 100 μL PBS) were intravenously injected into the mice. After 6, 12, and 24 h, coelenterazine (400 ng/100 μL in PBS) was intravenously injected into the mice, and the bioluminescent signal was immediately measured. Later, to evaluate the biodistribution of monobodies ex vivo, tumors and other organs were collected and analyzed using the IVIS Lumina imaging system (PerkinElmer, Waltham, MA, USA). The tumor area signals were calculated, and corresponding photon signals were quantified.

### 2.11. Statistical Analysis

All experiments were done in triplicate (*n* = 3) and the values from all experiments are expressed as the mean ± standard deviation (SD). Statistical analysis was done with two-way ANOVA using GraphPad Prism 5.0. *p*-values < 0.05 (significant), *p* < 0.01 (very significant), and *p* < 0.001 (extremely significant) are indicated on the graphs using the symbols *, **, and ****, respectively.

## 3. Results

### 3.1. Translocation of CRT to the Plasma Membrane after Treatment with Anticancer Drugs That Induce ICD

The anticancer agents MTX and DOX are known to cause the translocation of CRT to the plasma membrane during the pre-apoptotic stage (ecto-CRT) and to induce ICD [8]. By contrast, GEM does not induce ecto-CRT during the pre-apoptotic stage even though it induces cell death. To evaluate ecto-CRT on the plasma membrane during pre-apoptosis, CT-26 and MC-38 cells were treated with these anticancer agents for 4 h (Figure 1). Through Western blot analysis of CT-26 plasma membrane fractions, after MTX and DOX treatment, ecto-CRT was significantly increased (2.04-fold and 2.14-fold, respectively) compared with non-treated controls (Figure 1A,B). In MC-38 cells as well, ecto-CRT significantly increased after MTX and DOX treatment (2.25-fold and 2.39-fold, respectively), compared with non-treated controls. However, the ecto-CRT level did not increase during pre-apoptosis in GEM-treated cells. This result was similar by flow cytometry analysis (Figure 1C,D) and by confocal immunofluorescence imaging (Figure S2). Moreover, DOX and MTX significantly increased the expression of ecto-CRT in HeLa and MDA-MB-231 cells (Figure S1). These data indicate that DOX and MTX induced ecto-CRT exposure on the plasma membrane in the early (pre-apoptotic) phase of ICD.

### 3.2. Design and Characterization of CRT-Binding Monobodies

**Figure 2:**
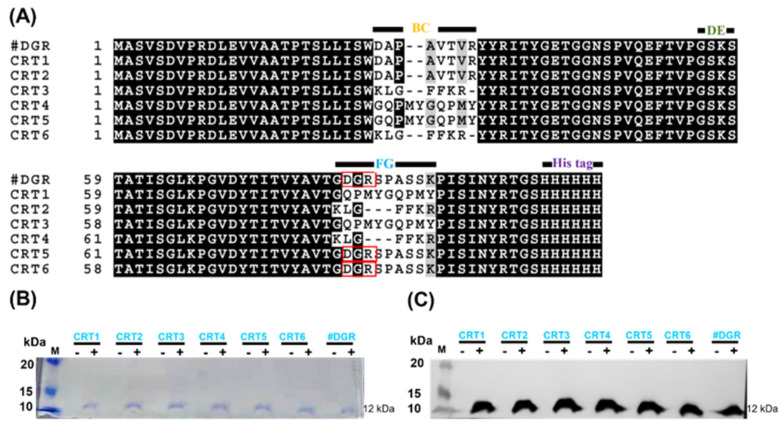
Engineering of calreticulin (CRT)-targeting monobodies. (**A**) Amino acid sequence alignment of monobodies (CRT1–CRT6). The sequences at the BC, DE, and FG loops are known to be related to target binding. The wild-type sequences at the BC and FG loops of FN3 were exchanged with those of the CRT-binding peptides Int-α (KLGFFKR) and Hep-I (GQPMYGQPMY). CRT5, CRT6, and FN3(DGR) contain DGR instead of RGD at the wild-type FG loop sequence (red boxes). At their C-termini, all monobodies carry His tags. Their assumed MWs are approximately 12 kDa. (**B**) Monobody expression in transformed *E. coli* BL21(DE3). With (+) or without (−) IPTG induction, bacteria transformed with pETh expression vectors were separated by SDS-PAGE. M, size marker (kDa). (**C**) Western blot analysis with an anti-His tag antibody. FN3(DGR) is indicated as #DGR.

Two peptides (Int-α and Hep-I) have been described to bind CRT [45,60,61]. Three loop sequences (BC, DE, and FG) of monobodies are responsible for target binding, and two of the loops (BC and FG) have a much longer length [62]. Therefore, we replaced the wild-type sequences with the Int-α and Hep-I peptide sequences at these loops. The engineered monobodies were named CRT1–CRT6. It should be noted that the RGD sequence at the FG loop was changed to DGR (a scrambled sequence of RGD) because this motif is responsible for integrin binding [63]. In this study, wild-type FN3 with a DGR sequence (named #DGR) was used instead of RGD as a negative control monobody. The amino acid sequences of all monobodies were clearly aligned, as shown in Figure 2A.

After the construction of expression vectors using the pET plasmid with a C-terminal His tag (pETh) [64], we established bacterial strains for monobody expression after their transformation into *E. coli* BL21(DE3). Monobodies were purified with an Ni-NTA affinity column from IPTG-induced bacterial cultures. The purified monobodies (MW, approximately 12 kDa) were analyzed by SDS-PAGE and by Western blotting against the His tag (Figure 2B,C; Table S2).

To determine which monobodies efficiently bound ecto-CRT, CT-26 cells were treated with 100 nM of each monobody after treatment with anticancer agents that induce ICD (DOX and MTX) and were analyzed by flow cytometry (Figure S3). Monobodies containing both peptide (Int-α and Hep I) sequences, CRT3 and CRT4, showed the highest binding to ecto-CRT in MTX-treated cells (Figure S3A,B). The mean fluorescence intensities (MFIs) were analyzed and presented in Figure S3 and Table S4. These results indicated that the CRT3 and CRT4 monobodies bound to ecto-CRT at least twice as strongly as the other monobodies. The results were similar in an analysis of DOX-treated cells (Figure S3C,D). These results indicate that the CRT-targeted peptide (Int-α and Hep-I) sequences synergistically increase the binding affinities of the ecto-CRT monobodies.

### 3.3. Characterization of Rluc8-Fused CRT-Binding Monobodies

**Figure 3:**
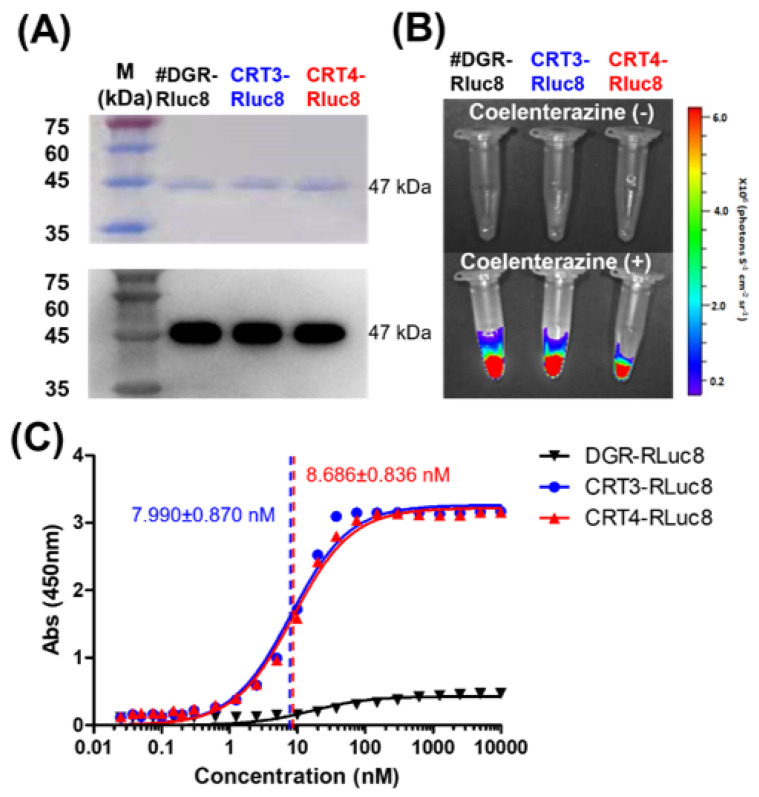
Purification and affinity measurements of Rluc8-fused monobodies. (**A**) Purification of Rluc8-fused monobodies. The monobodies expressed in *E. coli* (47 kDa) were purified with affinity chromatography, separated by SDS-PAGE (stained with Coomassie dye), and verified by Western blot analysis (anti-His tag antibody). (**B**) Luciferase assay with Rluc8-fused monobodies. After adding coelenterazine, the bioluminescence of the monobodies was measured with an IVIS imaging system. (**C**) Binding affinity measurements of monobodies using enzyme-linked immunosorbent assay (ELISA). Various concentrations of Rluc8-fused monobodies (0–10 μM) were coated onto the wells of an ELISA plate and 10 μM rCRT was added to each well. rCRT bound to monobodies was detected with an anti-CRT antibody. The colorimetric reaction represents the amount of rCRT bound to monobodies. Absorbance values in were plotted against the monobody concentrations (logarithmic scale).

Based on the above results, CRT-binding monobodies fused with C-terminal Rluc8 (CRT3-Rluc8 and CRT4-Rluc8, respectively) were generated and purified from *E. coli* by large-scale affinity chromatography. The purified Rluc8-fused monobodies had MWs of approximately 47 kDa (Figure 3A; Table S3). These monobodies showed strong bioluminescent signals in the presence of coelenterazine (Figure 3B).

The Kd values of CRT3-Rluc8 and CRT4-Rluc8 were measured by non-competitive ELISA assay. This assay results in a colorimetric reaction upon the addition of a substrate that correlates with the monobody concentration. Kd values of CRT3-Rluc8 and CRT4-Rluc8 (7.990 ± 0.870 nM and 8.686 ± 0.836 nM) showed efficient CRT binding (Figure 3C). These values were comparable to the affinities of other monobody proteins and typical antibodies [64,65,66,67,68], further demonstrating that the engineered CRT3 and CRT4 monobodies had strong binding affinities for CRT.

**Figure 4:**
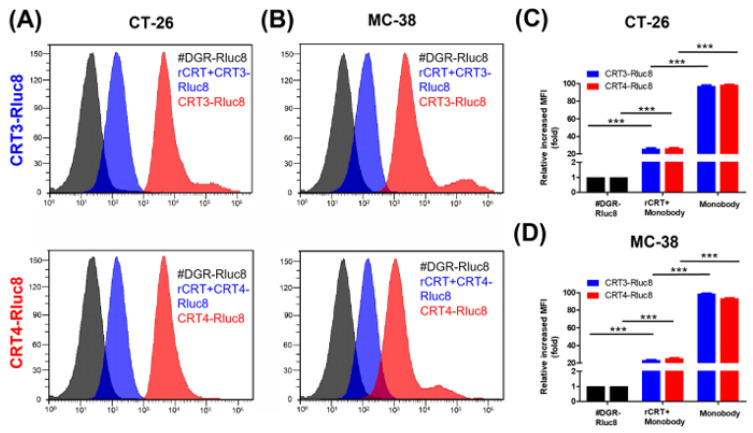
Monobodies specifically bound to ecto-CRT of cancer cells undergoing ICD. Cancer cells were treated with DOX and recombinant CRT (rCRT), and stained with Rluc8-fused monobodies. After washing, the cells were analyzed for monobody binding by flow cytometry. (**A**) Flow cytometry of CT-26 cells with exposed ecto-CRT mixed with rCRT. (**B**) Flow cytometry of MC-38 with exposed ecto-CRT mixed with rCRT. (**C**,**D**) Quantitation of (**A**) and (**B**). MFI levels of the monobodies bound to ecto-CRT were calculated relative to #DGR-Rluc8. Data are shown as the mean ± standard error (*n* = 3). *** *p* < 0.001.

**Figure 5:**
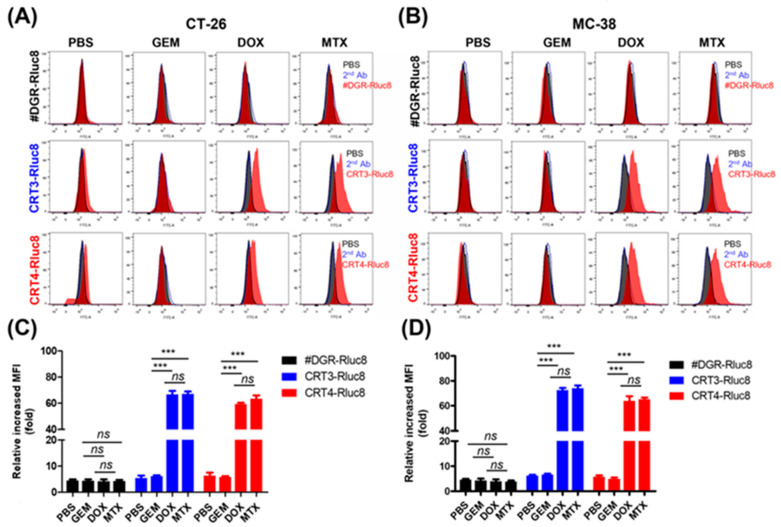
Rluc8-fused monobodies efficiently bound cancer cells with exposed ecto-CRT. Cancer cells (CT-26 and MC-38) were treated with anticancer agents (DOX, MTX, or GEM) for 4 h. Then, cells were stained with monobodies and analyzed by flow cytometry. (**A**,**C**) Flow cytometry of CT-26 cells. (**B**,**D**) Flow cytometry of MC-38 cells. PBS as negative control. Second Ab is used as control of the background fluorescence signals. The increase in MFI is shown relative to the respective control groups. Data show the mean ± standard error (*n* = 3). *** *p* < 0.001, and *ns* = non-significant.

**Figure 6:**
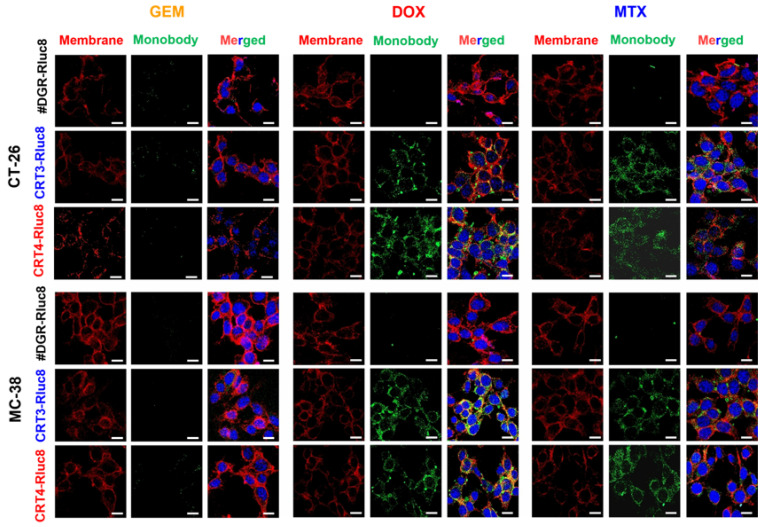
Immunofluorescence imaging analysis of the binding of Rluc8-fused monobodies to ecto-CRT in cancer cells treated with anticancer drugs. CT-26 and MC-38 cells treated with anticancer agents for 4 h were stained with ecto-CRT monobodies (CRT-3-Rluc8, CRT-4-Rluc8, and #DGR-Rluc8) and a secondary antibody (**green**) and observed by confocal microscopy (40×, magnification). The cell membranes were stained with fluorochrome-labeled wheat germ agglutinin (WGA) (**red**). Nuclei was stained with DAPI (**blue**). Scale bar represents 50 µm.

To verify that the Rluc8-fused monobodies specifically recognize ecto-CRT, competitive binding analysis of the monobodies was performed by flow cytometry using cells treated with anticancer agents and rCRT (Figure 4). In CT-26 and MC-38 cells treated with DOX, the MFI levels of the monobodies decreased about 73% in the presence of rCRT. FN3(DGR)-Rluc8 (#DGR-Rluc8) was used as a negative control for this experiment. Similar results were observed in HeLa and MDA-MB-231 cells (Figure S4). In addition, immunofluorescence imaging analysis of anticancer drug–treated cells was performed using an anti-CRT antibody and Rluc8-fused monobodies against ecto-CRT. As shown in Figure S2, MTX and DOX but not GEM and PBS expressed ecto-CRT on the cell surface during ICD. As expected, CRT3-Rluc8 and CRT4-Rluc8 monobodies efficiently bound to CT-26 and MC38 cells treated with DOX and MTX, but not GEM, by both flow cytometry and immunofluorescence imaging analyses (Figure 5 and Figure 6). Similar results were observed in HeLa and MDA-MB-231 cells treated with MTX and DOX (Figures S5 and S6). This clearly indicates that the CRT3 and CRT4 monobodies bind with high affinity to ecto-CRT expressed on cancer cells undergoing ICD [10,14,69].

### 3.4. In Vivo Imaging with Rluc8-Fused Monobodies in Animal Models

**Figure 7:**
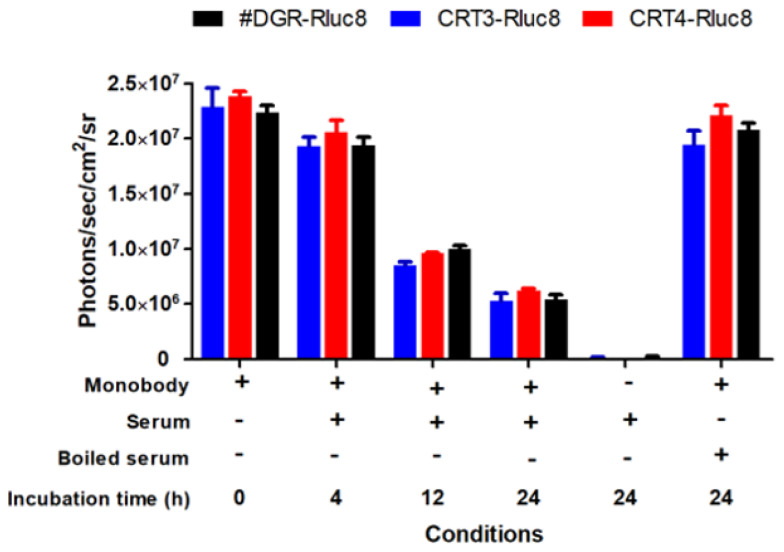
Stability of Rluc8-fused monobodies in serum. The monobodies were mixed with mouse serum and incubated at 37 °C for the indicated times. The luciferase bioluminescence activity of the monobodies was measured with an IVIS imaging system after adding coelenterazine (40 µg/mL). Data represent the mean ± standard error (*n* = 3).

To assess the efficacy of Rluc8-fused monobodies as in vivo imaging candidates, the stability of the monobodies was first measured in mouse serum after incubation at 37 °C (Figure 7). CRT3-Rluc8, CRT4-Rluc8, and #DGR-Rluc8 showed similar photon counts at each time-point, with a maximum value of 2.3 × 10^7^ photons/sec/cm^2^/sr. Their luciferase activities decreased to approximately 40% of the control after 12 h and to 24% after 24 h. This stability data is similar to that of other Rluc8-fused monobodies in mouse serum [52].

Next, we assessed therapeutic effects of anticancer agents in tumor-bearing mice in vivo. After the tumor volumes reached 100 mm^3^, the mice were administered MTX, DOX, or GEM (Figures S7 and S8). All the anticancer agents significantly and comparably suppressed tumor growth in CT-26 and MC-38 tumor-bearing mice. These data confirm that the anticancer agents at the administered doses had equivalent effects on the inhibition of tumor growth in mice.

**Figure 8:**
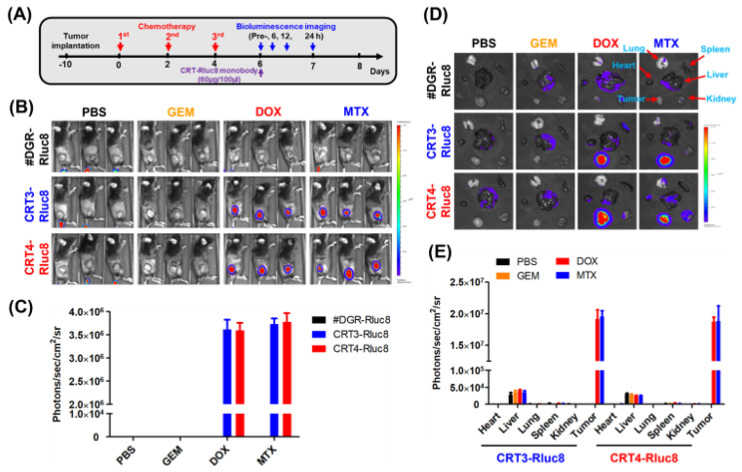
Ecto-CRT imaging with Rluc8-fusedmonobodies in tumor-bearing mice after anticancer drug treatment. (**A**) Scheme of anticancer drug administration and imaging conditions. The anticancer drugs were administrated to tumor-bearing mice (*n* = 3; three injections, every other day). Two days after the last drug treatment, engineered monobodies were intravenously injected into the mice. The bioluminescence imaging analysis was performed at the indicated time-points, after coelenterazine administration. (B) In vivo bioluminescence imaging analysis (12 h) with monobodies (#DGR-Rluc8, CRT3-Rluc8 and CRT4-Rluc8) in MC-38 tumor-bearing C57BL/6 mice under immunogenic (DOX and MTX) and non-immunogenic (GEM) anticancer treatments. (**C**) Quantification analysis of (**B**). Quantification analysis was not done in mice treated with GEM or PBS because the bioluminescence was below the limit of detection. (**D**) Ex vivo monobody biodistribution in tissues. Various tissues and organs were obtained from mice treated with monobodies and imaging analysis was done ex vivo after 12 h. (**E**) Quantification analysis of (**D**). Data represent the mean ± standard error (*n* = 3).

Finally, the targeting potential of the monobodies as in vivo diagnostic agents for the detection of ICD during chemotherapy was assessed. To do this, CRT3-Rluc8 and CRT4-Rluc8 (60 ug per mouse) were injected via the tail vein into MC-38 tumor-bearing mice pre-treated with anticancer agents (Figure 8). The bioluminescence signals from the injected monobodies were measured for 24 h (Figure 8A). The #DGR-Rluc8-injected groups did not show any bioluminescence signal in mice receiving any anticancer treatment. By contrast, significant signals were detected at 6 h, peaked at 12 h, and disappeared at 24 h in mice treated with DOX or MTX, but not GEM, after the injection of CRT3-Rluc8 or CRT4-Rluc8 (Figure 8B,C; Figure S9). Next, we measured the monobody biodistribution in mouse tissues at 12 h after the monobody injection (Figure 8D,E). The bioluminescence signals from Rluc8-fused monobodies were measured in dissected organs using the IVIS imaging system. Strong signals were observed in the tumors of mice treated with DOX and MTX but not GEM after monobody injection. The signal in #DGR-Rluc8-injected tumors was below the limit of detection. Similar results were obtained in CT-26 tumor-bearing mice (Figure S10). These results clearly indicate that the engineered CRT3 and CRT4 monobodies specifically bound to ecto-CRT during ICD. These monobodies can therefore be considered potential theranostic imaging candidates for the early detection of ICD during anticancer agents’ treatment.

## 4. Discussion

As shown in numerous previous reports, exposure of CRT at the cell surface during pre-apoptosis is an important hallmark of ICD [70]. This finding may enable us to predict the efficacy of ICD-inducing anticancer agents at early time-points [71]. In a recent report, an ^18^F-labeled CRT-targeted Int-α peptide showed promise as an ecto-CRT imaging agent in dying tumor cells during ICD-inducing anticancer drug treatment in vivo [8]. Another report showed that gold nanoclusters conjugated to an anti-CRT antibody could be used as a probe for near-infrared imaging to detect ecto-CRT in HT-29 colorectal cancer cells [72]. Our monobodies bound to CRT with a binding affinity comparable with that of the antibody and were more stable in serum than the peptides. Upon utilizing this CRT, targeting monobodies to detect ecto-CRT during chemotherapy would envisage the role in the activation of host-relevant innate and adaptive anticancer immune responses to dying cancer cells, which could further regulate ICD. These benefits suggest the possibility that the monobodies could be used clinically to detect ecto-CRT during chemotherapy administration.

Short tumor-binding peptides are considered a promising alternative to monoclonal antibodies as they can efficiently reach tumor targets due to their small molecular size and high binding affinity [73]. However, their clinical application as diagnostic agents are likely to be limited because peptides are sensitive to degradation by proteases in the circulation, resulting in reduced target binding. This limitation can be overcome by developing biomolecules with both a stable structure resistant to proteolysis and high-affinity target-binding sequences. Kadonosono, T. et.al., reported that peptides grafted into a particular site of a protein scaffold showed improved target affinity and resistance to proteolysis [74]. The FN3 domain has a stable structure and has been used as a protein scaffold for monobodies [25,75]. Yimchuen W. et.al demonstrated that monobodies grafted with HER2-targeting peptide sequences showed improved resistance to proteolysis and specific binding to HER2 [76]. In this study, we successfully developed CRT-binding monobodies by grafting CRT-binding peptide sequences (Int-α and Hep-I) onto the FN3 domain. Monobodies with single peptide sequences showed similar binding to ecto-CRT on the surface of cancer cells treated with ICD-inducing anticancer drugs (MTX and DOX) (Figure S3; Table S4). However, monobodies grafted with both peptide sequences (CRT3 and CRT4) bound to ecto-CRT more strongly than the monobodies with single peptide sequences. Because the two peptides bind to different sites of CRT, these data indicate that the binding affinities of the CRT3 and CRT4 monobodies were additively increased by the inclusion of both peptides. Consistent with this, the Kd values of CRT3 and CRT4 were similar (approximately 8 nM). These two monobodies showed similar stability in serum (an approximately 12 h half-life). The stability of CRT3 and CRT4 in serum is similar to the reported stability of monobodies against other targets [52]. Therefore, our peptide-grafted monobodies showed the expected high affinity and stability. Although our monobodies bound to ecto-CRT with a sufficiently high affinity to be useful as in vivo probes, it is expected that higher-affinity CRT-specific monobodies could be isolated if a library is made from these CRT monobody genes.

CRT monobodies could be applied as a therapeutic agent. ICD is known to occur during radio- and immunotherapies as well as chemotherapy [77]. If CRT monobodies fused with therapeutic biomolecules are developed and administered to tumor-bearing animals treated with such therapies, the therapeutic efficacy is expected to be enhanced. In models of tumor therapy using bacteria, various therapeutic biomolecules additively enhanced the therapeutic efficacy of the bacteria itself [78,79,80]. The expression and purification of Rluc8-fused monobodies in *E. coli* demonstrates the possibility that monobodies coupled with therapeutic biomolecules could be engineered.

## 5. Conclusions

Taken together, our data clearly demonstrate the functional properties of engineered CRT-targeting monobodies to detect ICD during cancer chemotherapy. This strategy of engineering novel monobodies using peptides may simplify the process required to generate high-affinity biomolecules for inaccessible or challenging targets.

## Data Availability

All the data in relevant to this manuscript are given in the supporting information. Any further date or part of the data’s can be obtained from the author upon reasonable request.

## Supplementary Materials

The following are available online at https://www.mdpi.com/article/10.3390/cancers13112801/s1, Figure S1: Measurement of ecto-CRT during immunogenic cell death (ICD) after anticancer drug treatment, Figure S2: Immunofluorescence imaging analysis of ecto-CRT on cells treated with anticancer agents, Figure S3: CRT3 and CRT4 monobodies bound efficiently to ecto-CRT on ICD-induced cancer cells, Figure S4: Monobodies specifically bound to ecto-CRT on ICD-induced cancer cells, Figure S5: Rluc8-fused monobodies bound strongly to ecto-CRT on cancer cells, Figure S6: Immunofluorescence imaging analysis with Rluc8-fused monobodies against ecto-CRT in cancer cells treated with anticancer drugs, Figure S7: Therapeutic efficacy of anticancer agents in CT-26 tumor-bearing mice, Figure S8: Therapeutic efficacy of anticancer agents in MC-38 tumor-bearing mice, Figure S9: Ecto-CRT imaging with Rluc8-fused monobodies at various time points in C57BL/6 tumor-bearing mice after anticancer treatments, Figure S10: Ecto-CRT imaging with Rluc8-fused monobodies at various time points in tumor-bearing mice after anticancer treatments in Balb/c mice, Figure S11: Full western blots. Table S1: Primers used in this study, Table S2: Quantification for Western blot analysis in Figure 2, Table S3: Quantification for Western blot analysis in Figure 3, Table S4: Quantification of mean fluorescence intensities (MFI) for Figure S3.
