# Supplementary material for: Engineering Calreticulin-Targeting Monobodies to Detect Immunogenic Cell Death in Cancer Chemotherapy
Source: Cancers (Basel). 2021 Jun 4;13(11):2801. doi: 10.3390/cancers13112801 (PMC8200062; doi:10.3390/cancers13112801)
Supplement: Supplementary file 1 [file cancers-13-02801-s001.zip › cancers-1202651-supplementary.pdf]

cancers MDPI

Supplementary Materials

Engineering Calreticulin-Targeting Monobodies to Detect Immunogenic Cell death in Cancer Chemotherapy

Ying Zhang [1,†], Ramar Thangam [1,2,†], Sung-Hwan You [1], Rukhsora D Sultonova [1], Akhil Venu [1], Jung-Joon Min [1,3,*] and Yeongjin Hong [1,3,*]

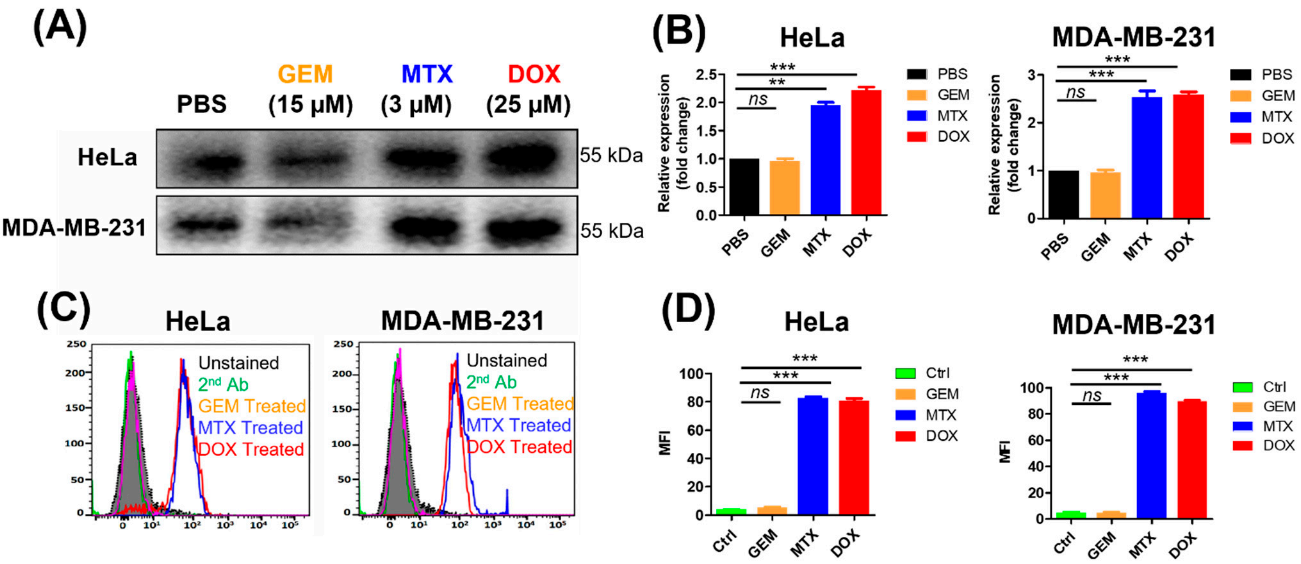

Figure S1. Measurement of ecto-CRT during immunogenic cell death (ICD) after anticancer drug treatment. The cancer cell lines HeLa and MDA-MB-231 were treated with anticancer agents at the indicated concentrations for 4 h, and ecto-CRT (CRT translocated from the ER to the plasma membrane) was measured at the pre-apoptotic stage. (A) Western blot analysis. The membrane fractions were isolated and separated by SDS-PAGE. Ecto-CRT was detected with an anti-CRT antibody. (B) Quantitation of western blotting data. The ecto-CRT detected in (A) was quantitated and the relative levels were plotted. (C) Flow cytometry analysis of ecto-CRT. Cells treated with anticancer agents were stained with an anti-CRT antibody. (D) Quantitation of flow cytometry. 2nd Ab is used as control to remove the background fluorescence signals. The mean fluorescence intensities (MFIs) of the samples shown in (C) were measured and the relative levels are plotted. Data represent the mean ± standard error (n = 3). ***P<0.001 and *ns*=non-significant. MTX, mitoxantrone; DOX, doxorubicin; GEM, gemcitabine.

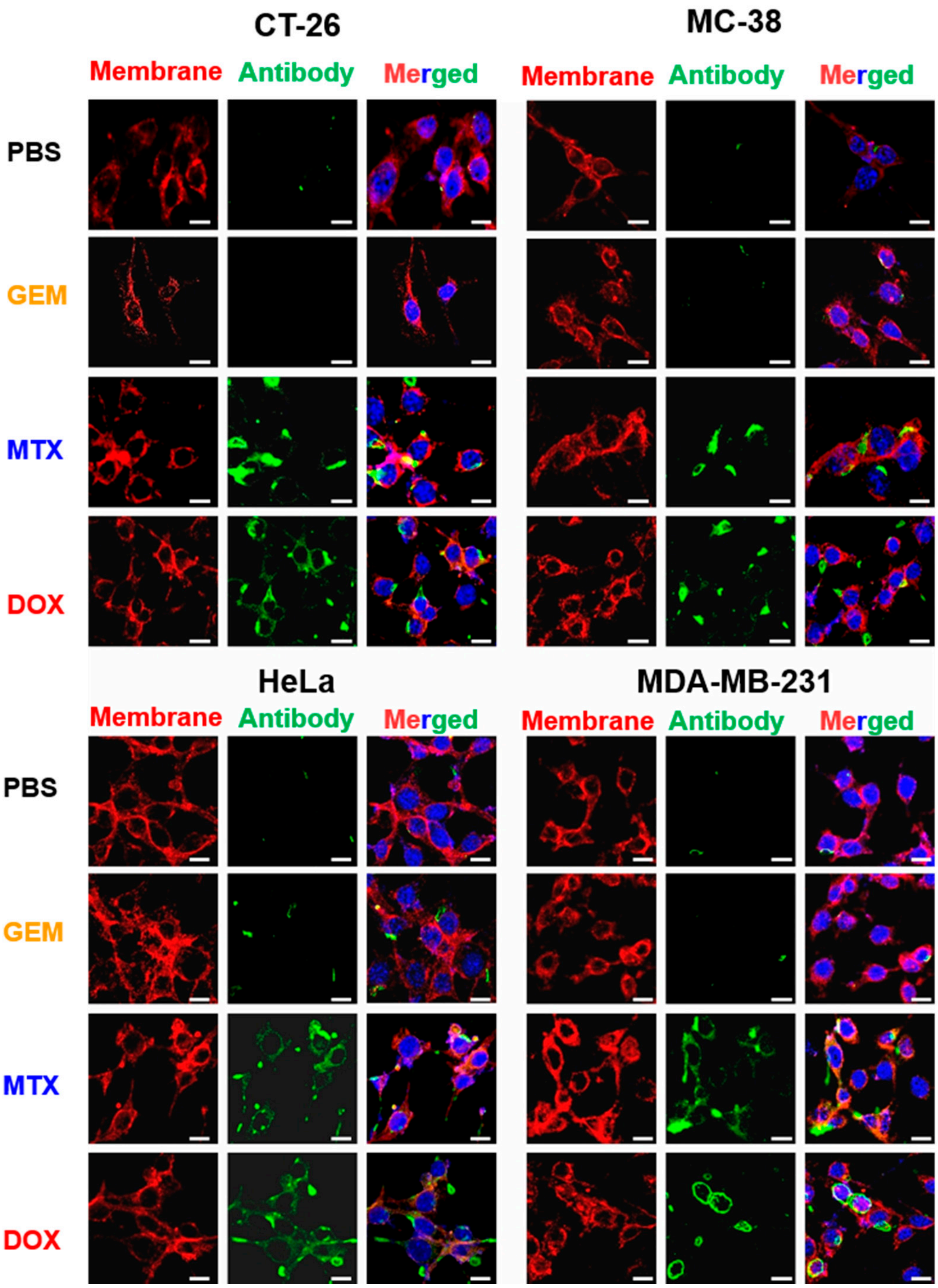

Figure S2. Immunofluorescence imaging analysis of ecto-CRT on cells treated with anticancer agents. Cancer cells (CT-26, MC-38, HeLa, and MDA-MB-231) treated with anticancer drugs (GEM, MTX, and DOX) for 4 h were stained with an anti-CRT antibody (green). Plasma membranes were also stained with wheat germ agglutinin (WGA) (red). Nuclei was stained with DAPI (blue). Magnification at 40×. Scale bar represents 50 μm.

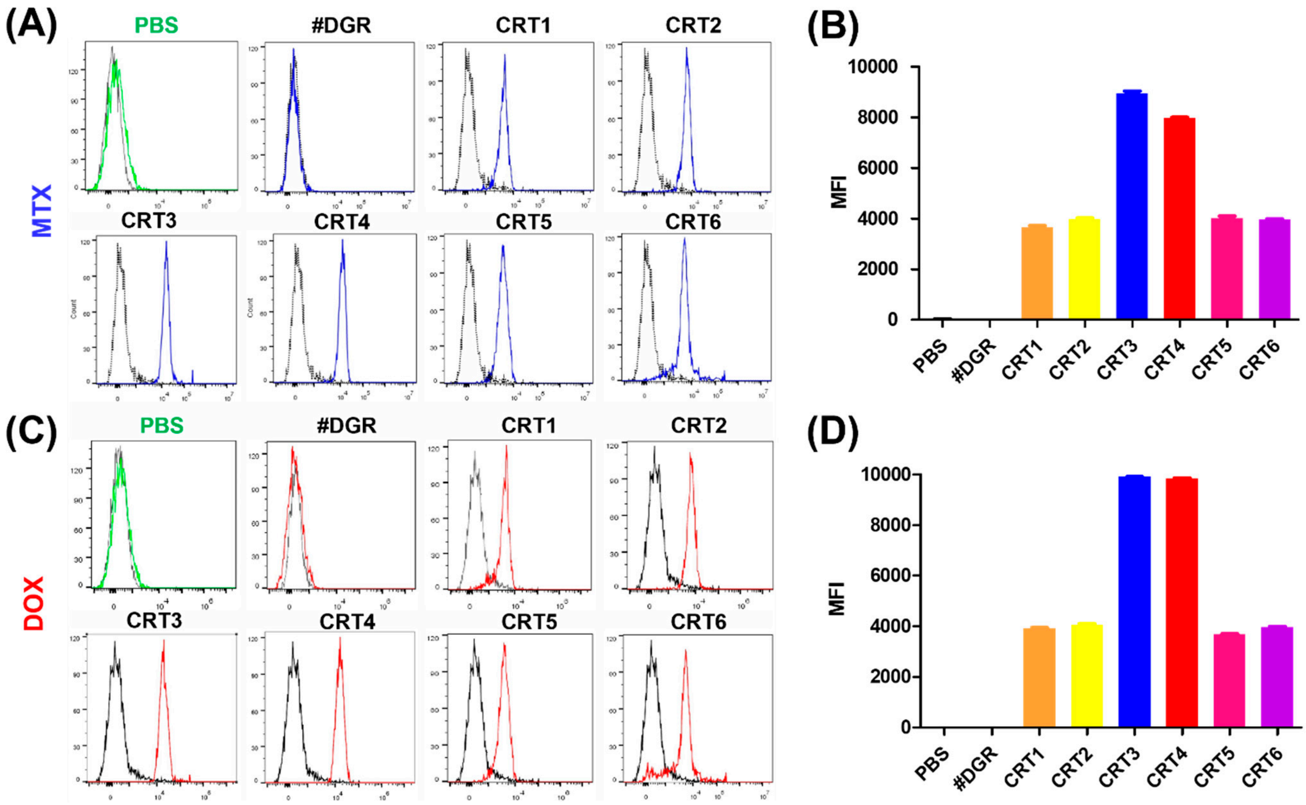

Figure S3. CRT3 and CRT4 monobodies bound efficiently to ecto-CRT on ICD-induced cancer cells. After anticancer treatments, cancer cells were incubated with the same amount of each monobody (100 nM). The cells were then sequentially labeled with an anti-His tag antibody and an Alexa Fluor 488-conjugated anti-mouse IgG antibody and the cells were analyzed by flow cytometry. (A and B) show the histograms of monobody binding to MTX-treated cells and its quantitation. (C and D) show the histograms of monobody binding to DOX-treated cells and its quantitation. The relative MFIs of monobody peaks (colored lines) were calculated against antibody only (black lines). Data represent the mean ± standard error (n = 3). FN3(DGR) is indicated as #DGR.

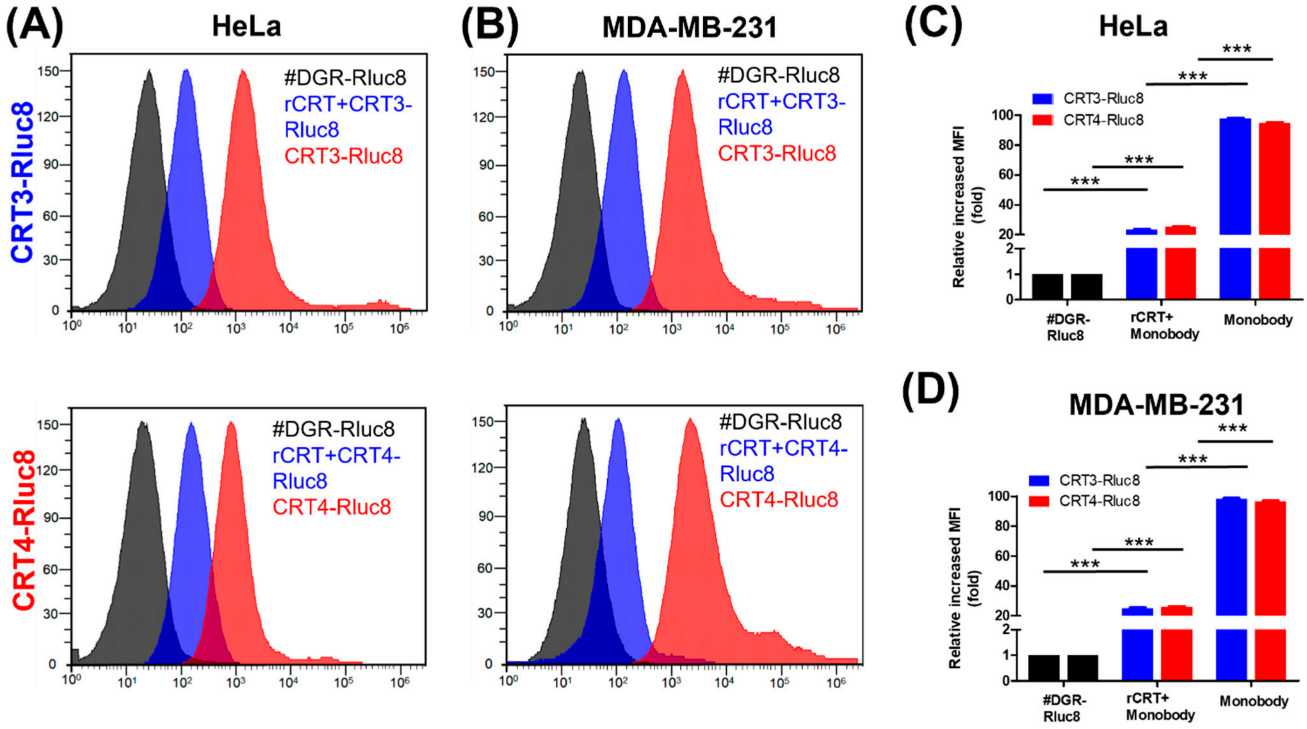

Figure S4. Monobodies specifically bound to ecto-CRT on ICD-induced cancer cells. A mixture of cancer cells treated with DOX and recombinant CRT (rCRT) was stained with Rluc8-fused CRT monobodies. After washing, the cells were analyzed by flow cytometry. (A) Flow cytometry of ecto-CRT on HeLa cells mixed with rCRT. (B) Flow cytometry of ecto-CRT on MDA-MB-231 cells mixed with rCRT. (C and D) Quantitation of A and B. The MFI values of the bound monobodies were calculated relative to the MFI of FN3(DGR)-Rluc8 (#DGR-Rluc8). Data represent the mean ± standard error (n = 3). ***P<0.001.

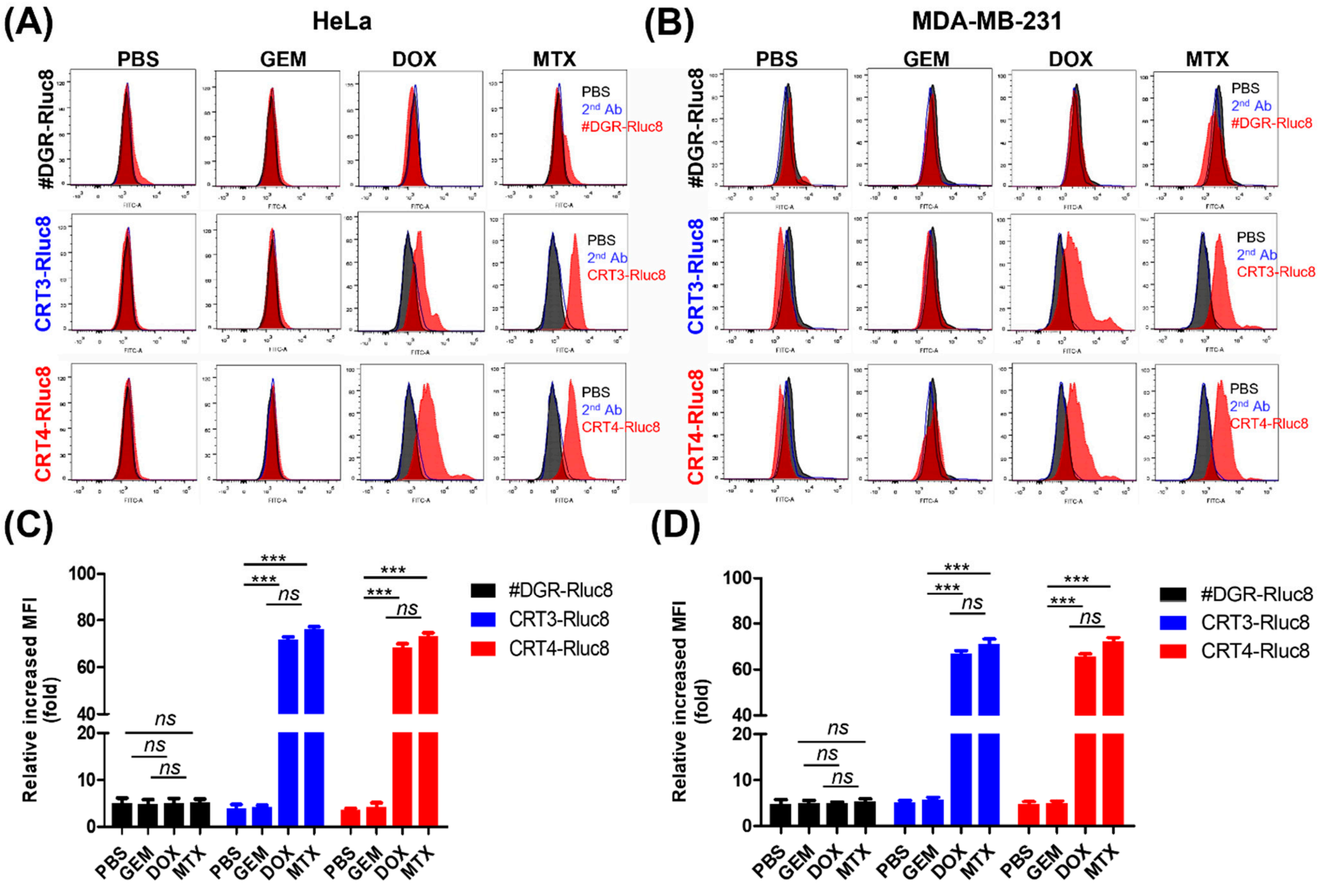

Figure S5. Rluc8-fused monobodies bound strongly to ecto-CRT on cancer cells. Cancer cell lines (HeLa and MDA-MB-231) were treated with anticancer agents (DOX, MTX, or GEM) for 4 h. Then, the cells were stained with monobodies and analyzed by flow cytometry. (A and C) Flow cytometry of HeLa cells and the corresponding quantification. (B and D) Flow cytometry of MDA-MB-231 cells and the corresponding quantification. PBS as negative control. 2nd Ab is used as control to remove the background fluorescence signals. Data represent the mean ± standard error (n = 3). ***P<0.001 and *ns*=non-significant.

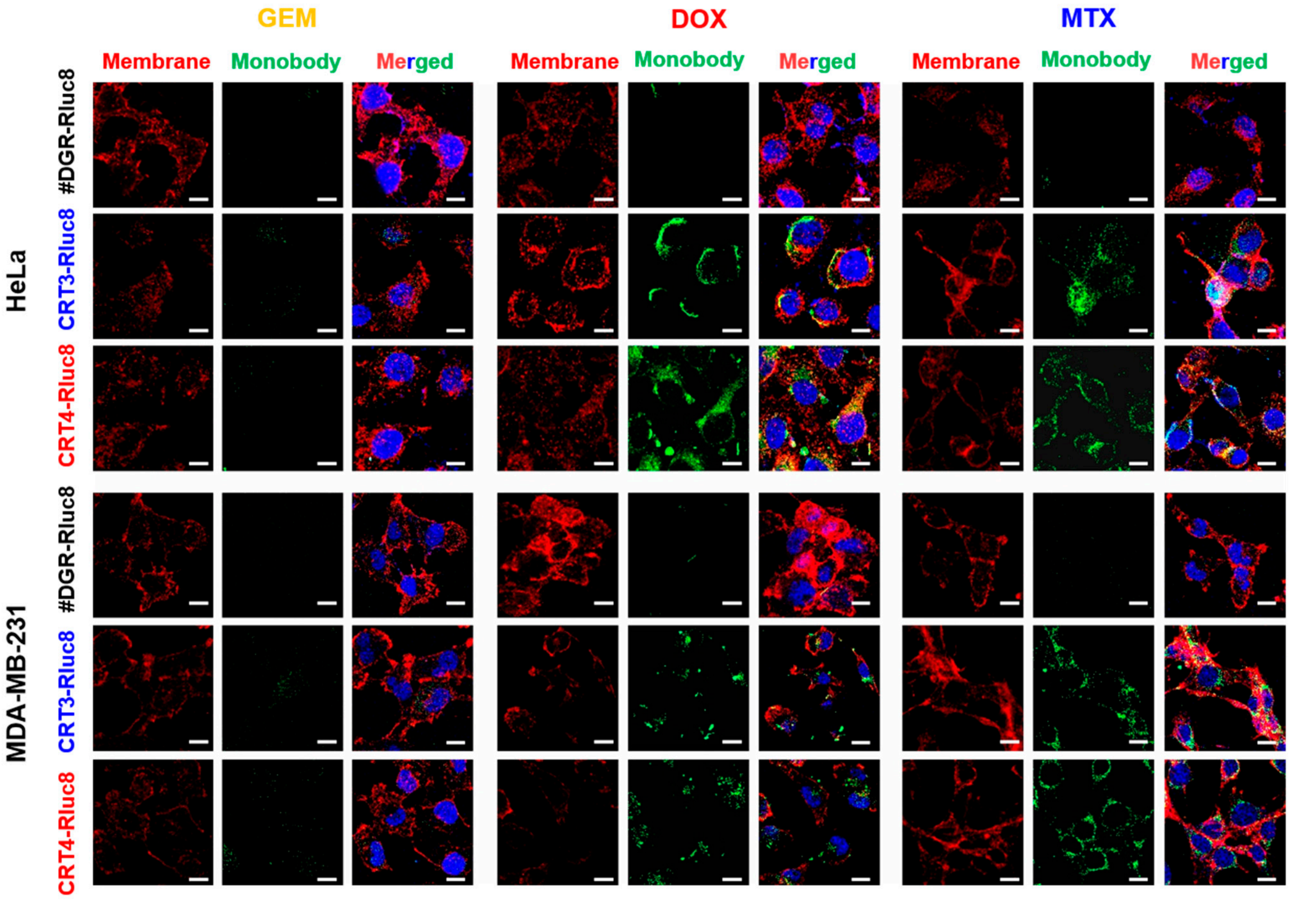

Figure S6. Immunofluorescence imaging analysis with Rluc8-fused monobodies against ecto-CRT in cancer cells treated with anticancer drugs. Cancer cells (HeLa and MDA-MB-231) were treated with anticancer agents for 4 h and stained with monobodies (green), and then observed by confocal microscopy (40×, magnification). Cell membranes were stained with wheat germ agglutinin (WGA) (red). Nuclei was stained with DAPI (blue). Scale bar represents 50 μm.

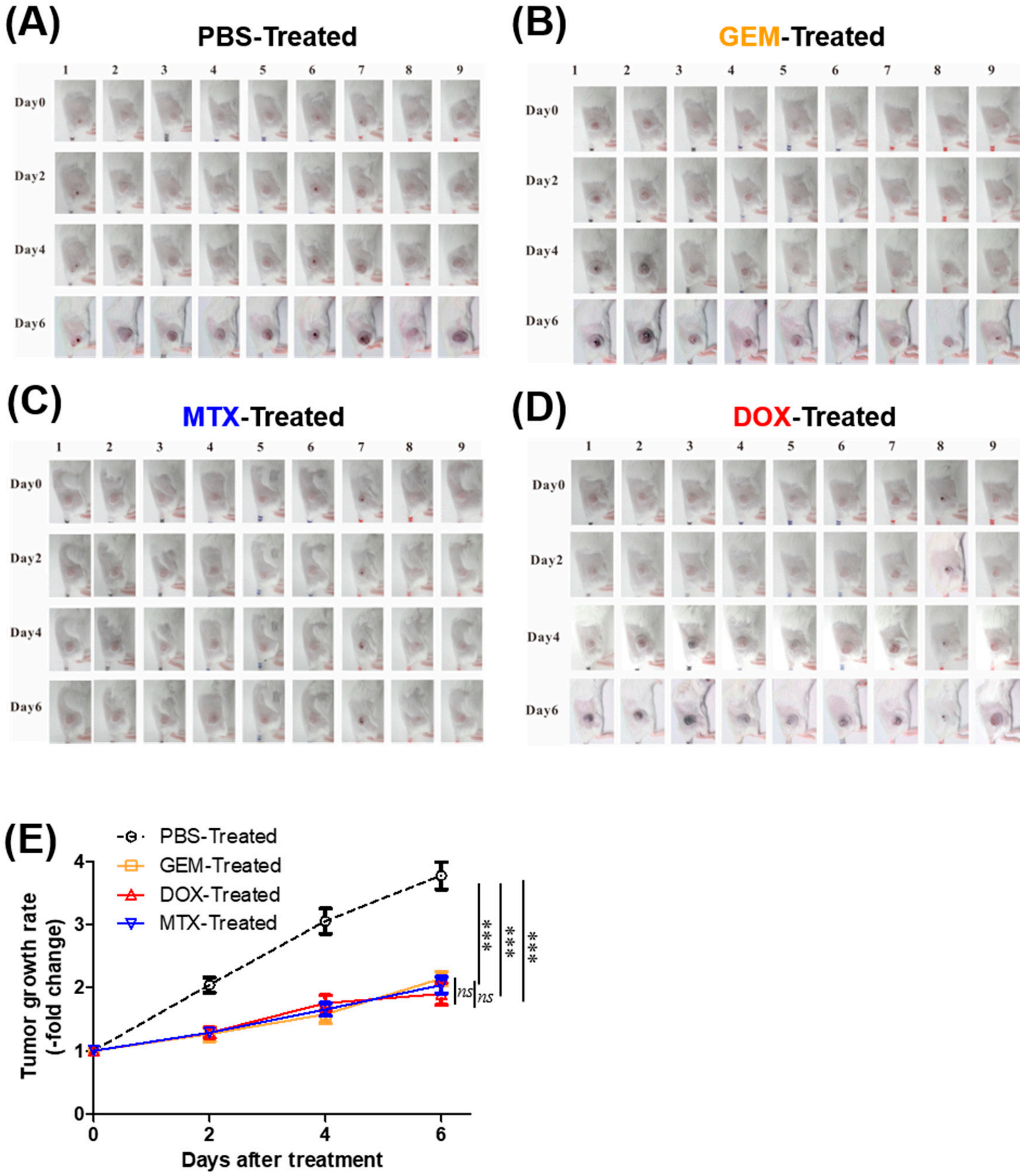

Figure S7. Therapeutic efficacy of anticancer agents in CT-26 tumor-bearing mice. Tumor-bearing BALB/c mice were treated with anticancer drugs (three injections at 2 day intervals) and tumor volumes were measured at the indicated time-points. (A) PBS treatment. (B) GEM treatment. (C) MTX treatment. (D) DOX treatment. (E) Quantification of tumor sizes. The tumor growth at each time-point is shown relative to the tumor size at day 0. Data represent the mean ± standard error (n = 9). ***P<0.001 and ns=non-significant.

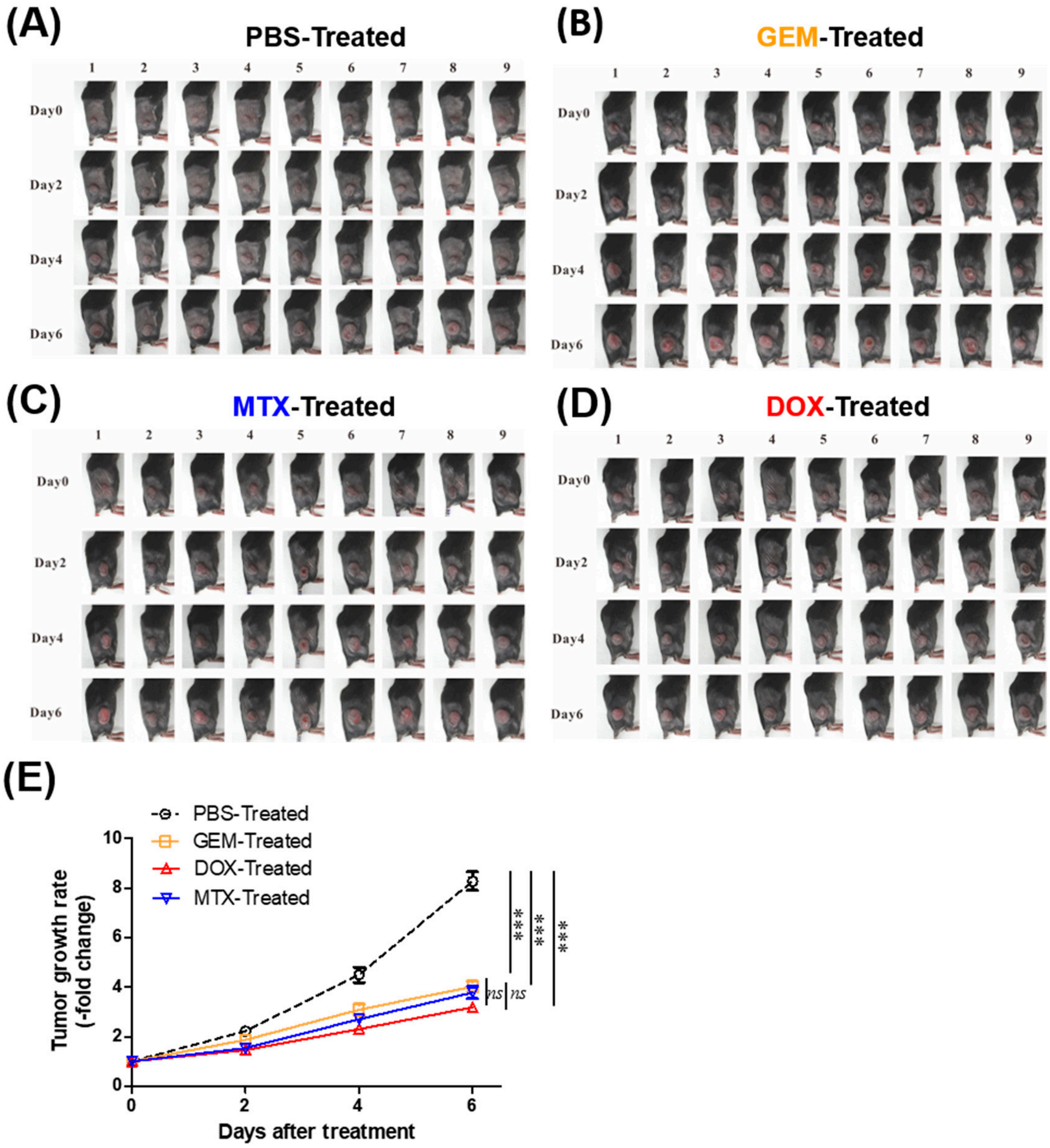

Figure S8. Therapeutic efficacy of anticancer agents in MC-38 tumor-bearing mice. Tumor-bearing C57BL/6 mice were treated with anticancer agents (three injections at 2 day intervals) and tumor volumes were measured at the indicated time-points. (A) PBS treatment. (B) GEM treatment. (C) MTX treatment. (D) DOX-treatment. (E) Quantification of tumor sizes. The tumor growth at each time-point is shown relative to the tumor size at day 0. Data represent the mean ± standard error (n = 9). ***P<0.001 and *ns*=non-significant.

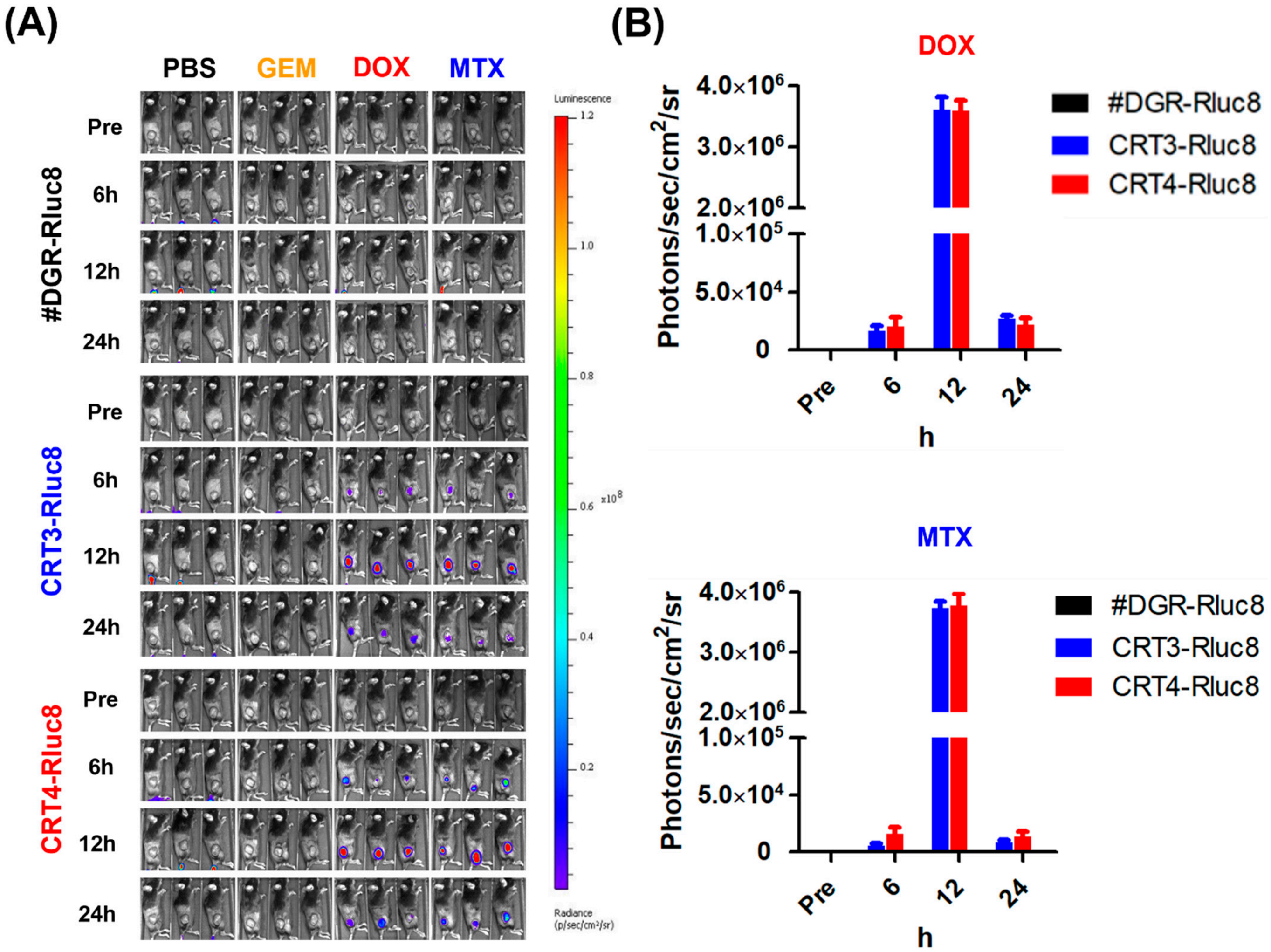

Figure S9. Ecto-CRT imaging with Rluc8-fused monobodies at various time points in C57BL/6 tumor-bearing mice after anticancer treatments. The anticancer agents were administrated to tumor-bearing C57BL/6 mice (n = 3; three injections at 2 day intervals). Two days after the last treatment, monobodies were intravenously injected into mice. Bioluminescence imaging analysis was performed before and at the indicated time-points (6, 12 and 24 h), after coelenterazine administration. (A) *In vivo* bioluminescence imaging analysis of CT-26 tumor-bearing mice. (B) Quantification of (A). Quantification was not done for mice treated with GEM (non-immunogenic agent) and PBS (as negative control) because the bioluminescence was below the limit of detection.

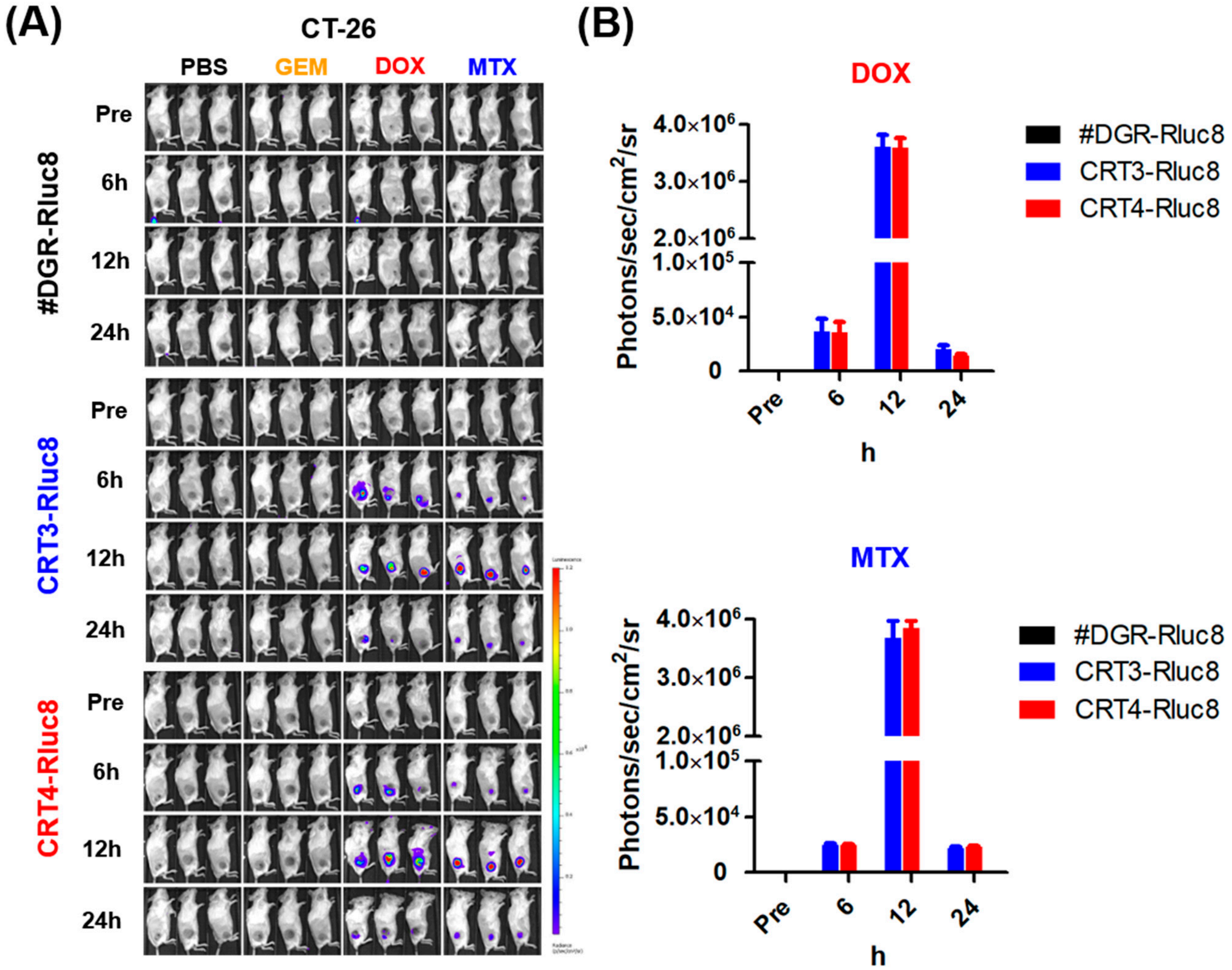

Figure S10. Ecto-CRT imaging with Rluc8-fused monobodies at various time points in tumor-bearing mice after anticancer treatments in Balb/c mice. The anticancer agents were administrated to tumor-bearing Balb/c mice (n = 3; three injections at 2 day intervals). Two days after the last treatment, monobodies were intravenously injected into mice. Bioluminescence imaging analysis was performed before and at the indicated time-points (6, 12 and 24 h), after coelenterazine administration. (A) *In vivo* bioluminescence imaging analysis of CT-26 tumor-bearing mice. (B) Quantification of (A). Quantification was not done for mice treated with GEM (non-immunogenic agent) and PBS (as negative control) because the bioluminescence was below the limit of detection.

Table S1. Primers used in this study.

Primer	Sequence
BC-Hep-1-R	5′-TTCTCCGTAAGTGATCCTGTAATACATTGGTTGACCATACATTGGTTGACCCCAGCTGATCAGTAGGCTGGTGGG
BC-Int-α-R	5′-TTCTCCGTAAGTGATCCTGTAATATCTTTTAAAAAAACCTAATTTCCAGCTGATCAGTAGGCTGGTGGG
FG-Hep-1-F	5′-ACCATCACTGTGTATGCTGTCACTGGTCAACCAATGTATGGTCAACCAATGTATCCAATTTCCATTAATTACCGAACA
FG-Int-α-F	5′-ACCATCACTGTGTATGCTGTCACTAAATTAGGTTTTTTTAAAAGACCAATTTCCATTAATTACCGAACA
BC-F	5′-CATATGGCTAGCGTTTCTGATGTTCCGAGGGACCTGGAAGTTGTTGCTGCGACCCCCACCAGCCTACTGATCAGCTGG
DE-F	5′-TATTACAGGATCACTTACGGAGAAACAGGAGGAAATAGCCCTGTCCAGGAGTTCACTGTGCCTGGGAGCAAGTCTACAGCTACC
DE-R	5′-AGTGACAGCATACACAGTGATGGTATAATCAACTCCAGGTTTAAGGCCGCTGATGGTAGCTGTAGACTTGCTCCCAGGCACAGT
94old-F	5'-TACATATGGCTAGCGTTTCTGATGTTCCGAG
94old-R	5'-TACTGAGTGGATCCTGTTCGGTAATTAATGGAAATTGG
Rluc8-F	5'-CGATGGGAATTCGCTTCCAAGGTGTACGA
Rluc8-R	5'-CAGGCCGGATCCAAGCTTCTGCTCGTTCT
T7-F	5′-TAATACGACTCACTATAGGGGAATTG
T7-R	5'-ATTTCGCGGGATCGAGATCTCGATC
FG-R	5'-TGTTCGGTAATTAATGGAAATTGG

Table S2. Quantification for western blot analysis in Figure 2.

Groups		Mean intensity value	Ratio of induction to w/o induction (+/-)
CRT1	w/o induction (-)	1282.555	145.721
	induction (+)	186895.179	
CRT2	w/o induction (-)	1282.555	142.758
	induction (+)	183095.399	
CRT3	w/o induction (-)	1257.369	152.211
	induction (+)	191385.721	
CRT4	w/o induction (-)	1251.254	155.195
	induction (+)	194187.884	
CRT5	w/o induction (-)	1214.23	156.591
	induction (+)	190137.547	
CRT6	w/o induction (-)	1224.148	140.572
	induction (+)	172080.765	
#DGR	w/o induction (-)	1231.521	136.186
	induction (+)	167715.524	

Table S3. Quantification for western blot analysis in Figure 3.

Monobody (purified 0.5ug)	Mean intensity value
#DGR-Rluc8	228341.491
CRT3-Rluc8	216952.234
CRT4-Rluc8	210073.452

Table S4. Quantification of mean fluorescence intensities (MFI) for Figure S3.

Groups	Mean fluorescence intensities (MFI)
PBS	33.93 ± 4.83
#DGR	36.57 ± 2.87
CRT1	3643.52 ± 133.01
CRT2	3971.64 ± 74.99
CRT3	8937.88 ± 151.17
CRT4	7951.06 ± 76.74
CRT5	4006.15 ± 148.44
CRT6	3942.38 ± 75.77
