# Supplementary figures and images for: Engineering Calreticulin-Targeting Monobodies to Detect Immunogenic Cell Death in Cancer Chemotherapy
Source: Cancers (Basel). 2021 Jun 4;13(11):2801. doi: 10.3390/cancers13112801 (PMC8200062; doi:10.3390/cancers13112801)

Figure 2B

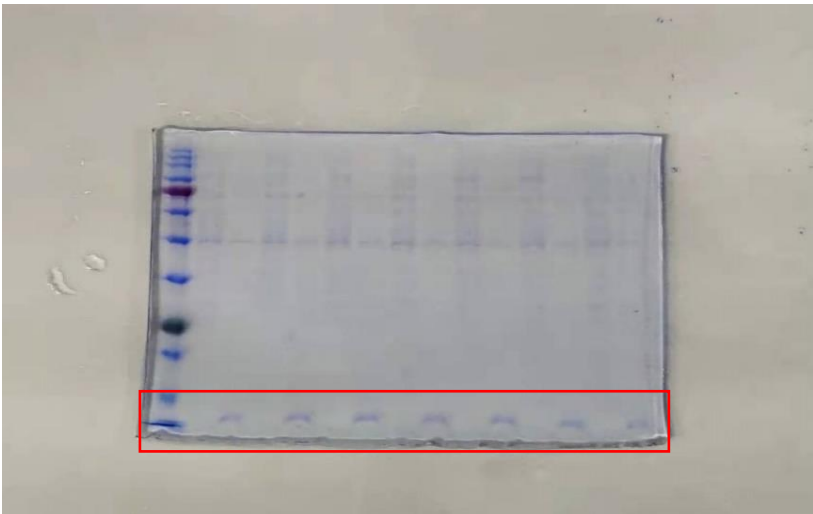

Figure 2C

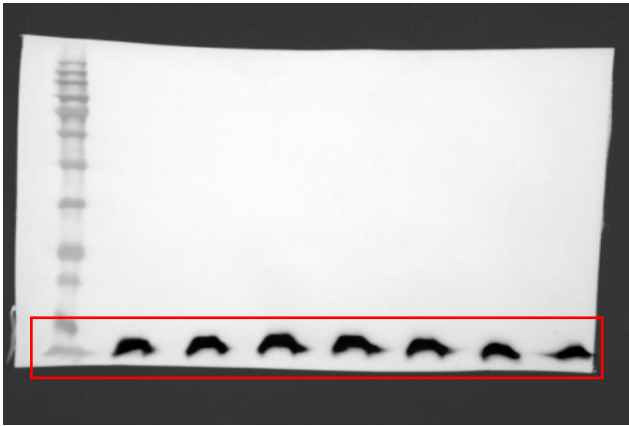

Figure 3A

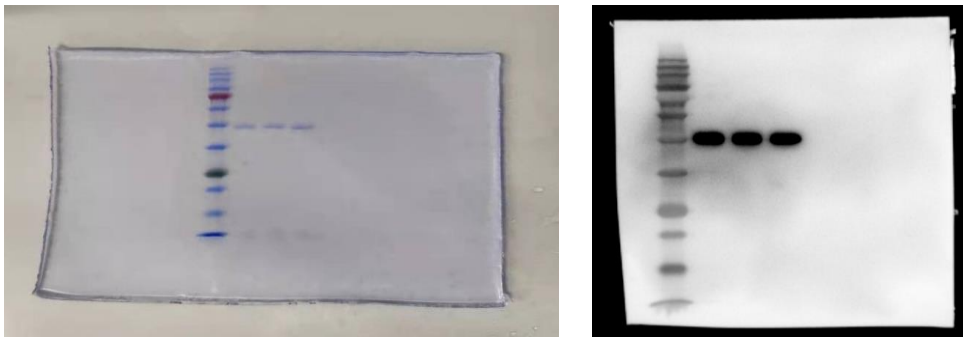

Figure 1

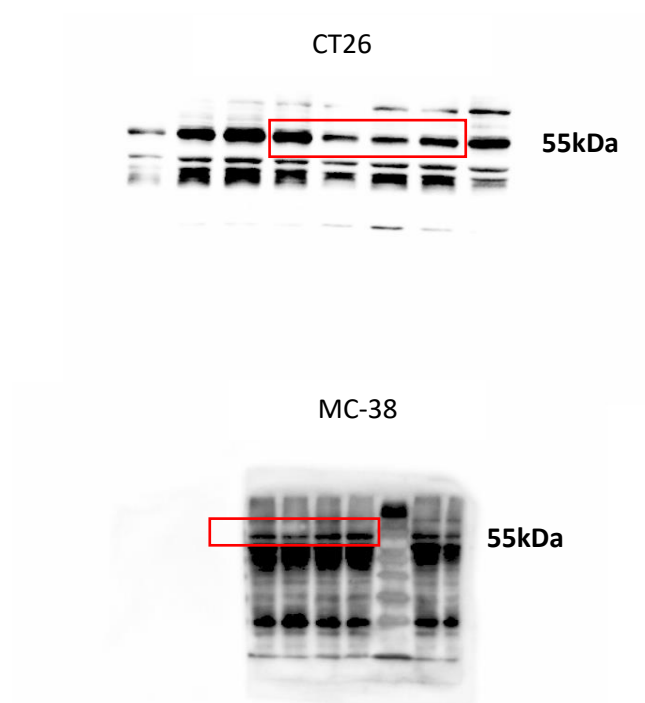

Figure S1

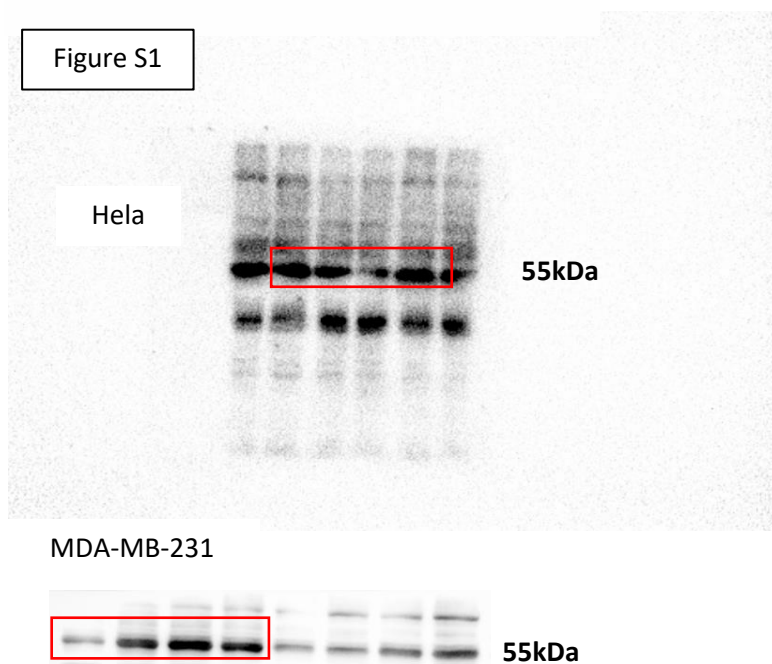

Supplement: Supplementary file 1 [file cancers-13-02801-s001.zip › Figure S11. Western Bot Images.pdf]
